# Construction of cellulose-utilizing *Escherichia coli* based on a secretable cellulase

**DOI:** 10.1186/s12934-015-0349-7

**Published:** 2015-10-09

**Authors:** Dongfang Gao, Yaqi Luan, Qian Wang, Quanfeng Liang, Qingsheng Qi

**Affiliations:** State Key Laboratory of Microbial Technology, Shandong University, Jinan, 250100 People’s Republic of China

**Keywords:** *Escherichia coli*, Cellulase, Extracellular expression, Cellulose, Poly (3-hydroxybutyrate)

## Abstract

**Background:**

The microbial conversion of plant biomass into value added products is an attractive option to address the impacts of petroleum dependency. The Gram-negative bacterium *Escherichia coli* is commonly used as host for the industrial production of various chemical products with a variety of sugars as carbon sources. However, this strain neither produces endogenous cellulose degradation enzymes nor secrets heterologous cellulases for its poor secretory capacity. Thus, a cellulolytic *E. coli* strain capable of growth on plant biomass would be the first step towards producing chemicals and fuels. We previously identified the catalytic domain of a cellulase (Cel-CD) and its N-terminal sequence (N20) that can serve as carriers for the efficient extracellular production of target enzymes. This finding suggested that cellulose-utilizing *E. coli* can be engineered with minimal heterologous enzymes.

**Results:**

In this study, a β-glucosidase (Tfu0937) was fused to Cel-CD and its N-terminal sequence respectively to obtain *E. coli* strains that were able to hydrolyze the cellulose. Recombinant strains were confirmed to use the amorphous cellulose as well as cellobiose as the sole carbon source for growth. Furthermore, both strains were engineered with poly (3-hydroxybutyrate) (PHB) synthesis pathway to demonstrate the production of biodegradable polyesters directly from cellulose materials without exogenously added cellulases. The yield of PHB reached 2.57–8.23 wt% content of cell dry weight directly from amorphous cellulose/cellobiose. Moreover, we found the Cel-CD and N20 secretion system can also be used for the extracellular production of other hydrolytic enzymes.

**Conclusions:**

This study suggested that a cellulose-utilizing *E. coli* was created based on a heterologous cellulase secretion system and can be used to produce biofuels and biochemicals directly from cellulose. This system also offers a platform for conversion of other abundant renewable biomass to biofuels and biorefinery products.

## Background

Plant biomass, a source of renewable and environmental friendly energy, has attracted much attention in recent years [1]. It has become an alternative candidate besides petroleum for the production of chemicals and biofuels because it is rich in fermentable sugar polymers [2, 3]. Plant biomass is mainly composed of three fractions: cellulose, hemicelluloses and lignin [4]. The proportion of these three fractions varies in different plants. However, the major constituent is cellulose which is a polymer of d-glucose linked by a β-1, 4-glycosidic bond, with cellobiose as the repeating unit [5, 6]. Numerous promising processes for various fuels and chemicals production have been developed to use both cellulose and cellobiose as carbon sources [7–9]. During the degradation of plant biomass, the endo-glucanase attacks cellulose and produces shorter cello-oligosaccharides. The exo-glucanase acts on the ends of the cellulose chains to produce mainly cellobiose, which is further degraded by β-glucosidase into glucose [10, 11]. Since this step needs expensive exogenous cellulose degradation enzymes, an efficient and cost effective process for the degradation and fermentation of plant biomass into commodity products is required [12]. Thus, incorporating enzyme generation, biomass degradation and product production into a single organism through engineering, could potentially avoid the cost of generating enzymes in the process of using cellulose [13].

The Gram-negative bacterium *Escherichia coli* has been widely used as a cell factory for the production of diverse chemicals because it is the best characterized model bacterium with many available expression and regulation tools [14]. Although *E. coli* can ferment a variety of sugars, including xylose, it is unable to degrade cellulose polymers [15]. The common laboratory strains of *E. coli* are poor secretors of proteins under normal culture conditions, which also limits its application in the extracellular production of cellulase [16]. Thus, a cellulolytic *E. coli* strain capable of growth on plant biomass would be the first step towards producing chemicals and fuels at a relative low cost. To solve this problem, researchers tried to fuse selected cellulases with carrier peptides for extracellular production or surface display [6, 17]. For example, Bokinsky et al. constructed a recombinant *E. coli* strain by fusion of the target protein with osmotically-inducible protein Y (OsmY), which can secrete xylanases and cellulases. This *E. coli* strain allows rapid and efficient growth on cellulose and hemicellulose pretreated with ionic liquids (IL) [13]. Tanaka and colleagues developed a cello-oligosaccharide assimilating *E. coli* strain by expressing β-glucosidases on the cell surface using a novel anchor protein [18]. All these researchers used a carrier that can guide the cellulolytic enzymes out of the cells. However, these commonly used carrier proteins are not involved in cellulose hydrolysis [13].

Polyhydroxybutyrates (PHB) are a kind of intracellular biopolymers accumulated by numerous microorganisms as carbon and energy storage materials during conditions of nutritional limitation in the presence of a carbon source excess [19]. It has been considered to be strong candidates for biodegradable polymer material because of its biodegradable and biocompatible properties [20]. A major drawback to the commercialization of PHB is their much higher production cost compared with petrochemical-based synthetic plastic materials [21].

In our previous study, we found that a catalytic domain of a cellulase (Cel-CD) and its N-terminus can be employed as carriers for extracellular production of recombinant proteins in large quantity, which suggested potential application for construction of cellulose-utilizing *E. coli* strain [22]. In this study, we fused a β-glucosidase (Tfu0937) from *Thermobifida fusc*a YX to Cel-CD. The fusion proteins digested the cellulose outside the host and the resulting glucose could be imported into the cells. Based on this, we set up a PHB production bioprocessing in *E. coli* strain using cellulose as the sole carbon source.

## Results

### Extracellular expression of Cel-CD in *E. coli* BL21 (DE3)

In previous research, we identified the catalytic domain of an alkaline cellulase with endo-beta-glycosidase activity (Cel-CD) from *Bacillus* sp., which can be secreted into the medium from recombinant *E. coli* BL21 (DE3) in large quantities. Both the Cel-CD and its N-terminal sequence were confirmed as carriers for the extracellular production of target proteins [22].

**Fig. 1:**
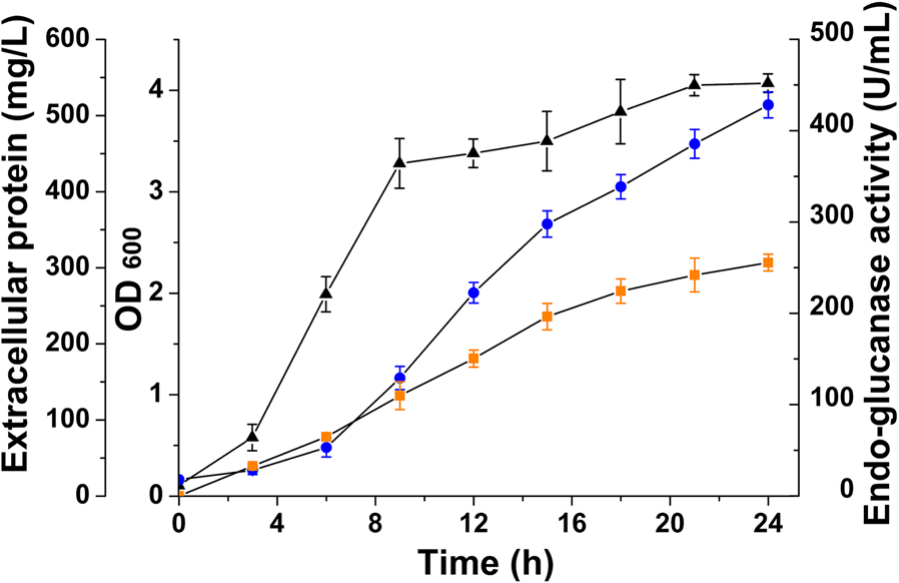
Cell density, accumulation of extracellular proteins and Cel-CD activity during cultivation. Cells were cultured in LB medium at 37 °C. *Circle markers* totally extracellular proteins in the culture medium; *triangle markers* growth curve of the Cel-CD expression strain; *square markers* the endo-glucanase activity of Cel-CD in the culture medium

In this study, the *cel*-*cd* gene was subcloned into the T7-driven plasmid pACYC-duet, generating pACYCDuet/cel. The recombinant *E. coli* BL21 (DE3) harboring the pACYCDuet/cel was analyzed with respect to its endo-glucanase activity (Fig. 1). The maximum extracellular protein concentration reached 513.84 mg/L and the maximum hydrolytic activity of Cel-CD towards carboxymethylcellulose (CMC) reached 255.48 U/mL, which was consistent with our previous results. The high extracellular endo-glucanase activity provided a possibility to engineer a recombinant strain that is able to completely digest cellulose to form glucose.

The substrate specificity of the extracellular Cel-CD was also examined by using two types of cellulosic materials, carboxymethylcellulose (CMC) and phosphoric acid swollen cellulose (PASC). Although Cel-CD showed high CMC hydrolysis activity, no PASC hydrolysis activity was detected (data not shown). This result indicated that Cel-CD has unique substrate specificity.

### Construction of the recombinant *E. coli* for glucose generation from cellulose

**Fig. 2:**
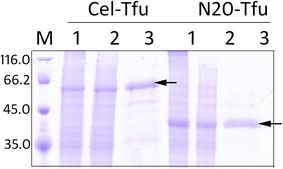
Secretion analysis of recombinant Cel-Tfu0937 and N20-Tfu0937. Cells were harvested by centrifugation after 16 h of induction and washed with 1 mL of 100 mM MOPS (pH 7.0) and separated on 12 % SDS-PAGE. Sample aliquots of 20 μL were loaded onto the gel. Molecular size markers are shown in kDa. *Lane M* is the marker; *Lane 1* is the whole cells; *Lane 2* is the supernatant of the disrupted cells; *Lane 3* is the released protein in the medium

As the soluble oligosaccharides that are produced by Cel-CD hydrolysis cannot be directly metabolized by *E. coli*, we fused the β-glucosidase (Tfu0937) from *Thermobifida fusca* to the C-terminus of Cel-CD [18]. The pN20-Tfu, which contains the N-terminal 20 amino acid residues (N20) of Cel-CD, was also constructed to investigate the secretion possibility of β-glucosidase and for the direct analysis of its activity. SDS-PAGE analysis showed that both Cel-Tfu and N20-Tfu were detected in the culture medium after 16 h of cultivation in LB medium, indicating they were successfully expressed (Fig. 2).

**Fig. 3:**
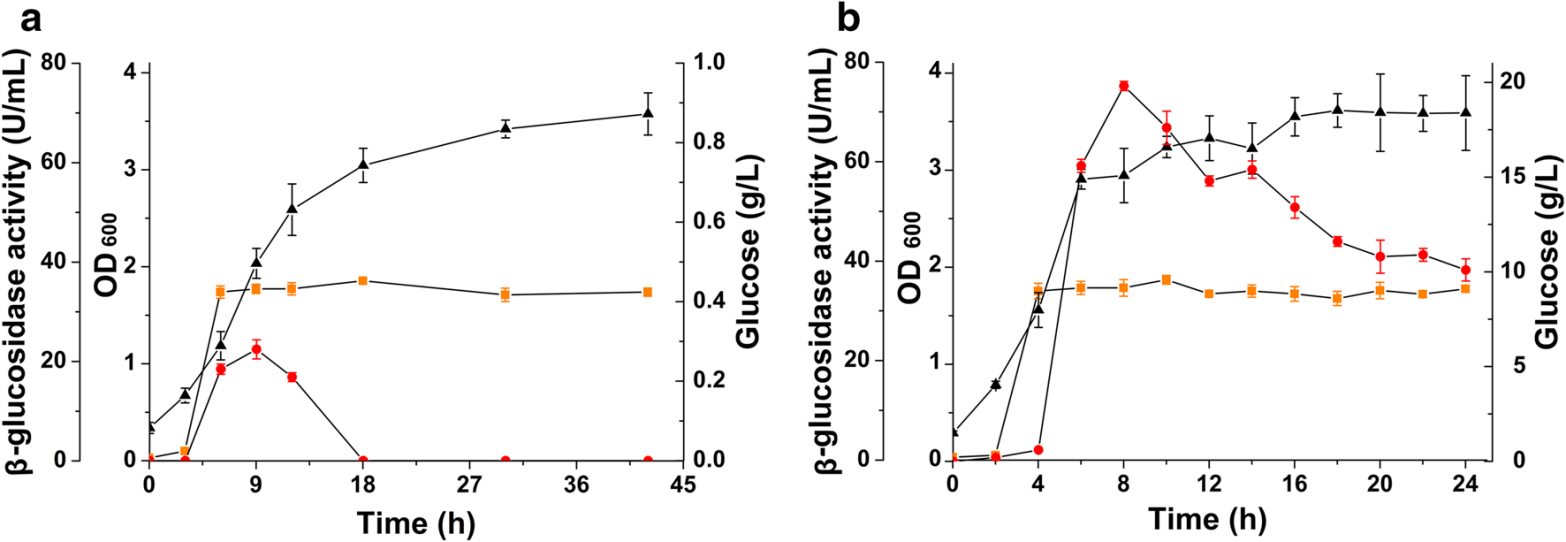
Growth curve, BGL activity and glucose accumulation of recombinant *E. coli*. The N20-Tfu expression strain was cultured in modified M9 medium supplemented with 20 g/L cellobiose as sole carbon source (**a**) and the Cel-Tfu expression strain supplemented with 20 g/L CMC (**b**). *Circle markers* glucose accumulation in the medium; *triangle markers* growth curve of the expression strains; *square markers* BGL activity of the secreted N20-Tfu and Cel-Tfu

**Fig. 4:**
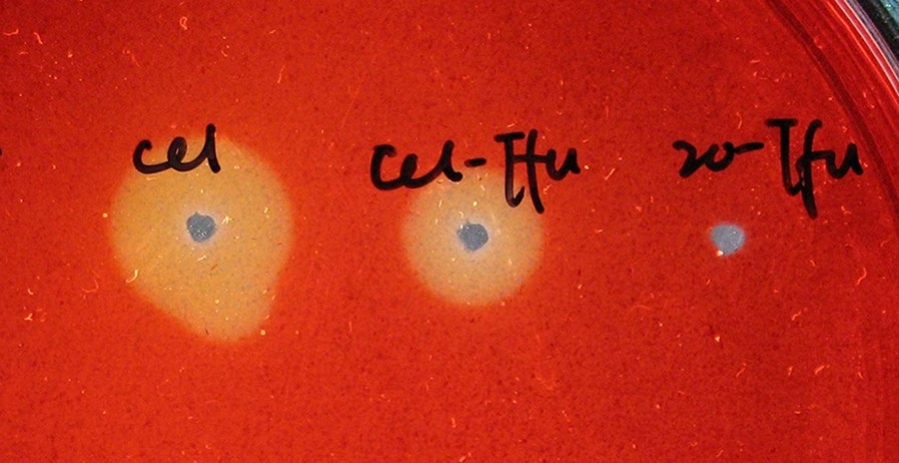
Activity analysis of Cel-CD in the medium. The endo-glucanase activities of the secreted Cel-Tfu and N20-Tfu were analyzed by the Congo red staining method

The BGL activity in the culture medium was quantitatively evaluated using *p*-nitrophenyl-β-d-glucopyranoside (pNGP) as the substrate (Fig. 3). After 24 h of fermentation, the activity of N20-Tfu and Cel-Tfu reached 38 and 37 U/mL in the culture medium, respectively, indicated that fusion with Cel-CD did not affect the secretion and activity of BGL. The endo-glucanase activity of Cel-Tfu in the medium was confirmed by Congo red staining as shown in Fig. 4 and its maximum hydrolytic activity towards carboxymethylcellulose (CMC) was detected, reaching 46.83 U/mL. These results clearly demonstrated that recombinant *E. coli* was able to actively secrete the fusion protein N20-Tfu and Cel-Tfu for biomass use.

In order to test whether the recombinant strains were able to utilize the biomass, these two *E. coli* strains were cultivated in modified M9 medium supplemented with 20 g/L cellobiose or cellulose (CMC) as sole carbon source, respectively. The cell growth and the glucose accumulation were also analyzed. When *E. coli* strain harboring pN20-Tfu cultured with cellobiose as the sole carbon source, the OD_600_ reached 3.91 after 8 h of cultivation, while the concentration of glucose reached 18.24 g/L (Fig. 3a). When *E. coli* strain harboring pCel-Tfu cultured with CMC as the sole carbon source, the glucose accumulation in the medium reached 0.36 g/L at 9 h and the final OD_600_ value was 3.57 (Fig. 3b). These results indicated that recombinant *E. coli* harboring cellulosic enzymes can grow and accumulate glucose when cellobiose or CMC used as the sole carbon source. Accumulation of glucose in the medium indicated that the recombinant strains have high cellulosic activity to support cell growth and product accumulation.

### PHB production from cellobiose and cellulose

To demonstrate the application of recombinant cellulolytic *E. coli*, a PHB biosynthesis pathway was constructed by introducing plasmid pBHR68, which contains acetoacetyl-coA (*phbB*), a β-ketothiolase (*phbA*) and an engineered PHB synthase (*phbC*) encoding gene. When 20 g/L cellobiose was used as the sole carbon source, the cellulolytic *E. coli* accumulated 1.71 g/L cell dry weight with a PHB yield of 0.14 g/L. The PHB content in the cell was 8.23 wt%. When the same strain was cultivated in 20 g/L glucose, a 2.12 g/L accumulated cell mass with a PHB yield of 0.30 g/L was obtained (Table 1). The PHB content was 14.00 wt%.

**Table 1:** PHB accumulation in recombinant *E. coli* harboring pN20-Tfu & pBHR68 and pCel-Tfu & pBHR68

Strain	Carbon source	CDW (g/L)	PHB content (wt%)	Total yield (g/L)
pN20-Tfu & pBHR68	Glucose	2.12 ± 0.10	14.24 ± 0.24	0.30 ± 0.02
	Cellobiose	1.71 ± 0.14	8.23 ± 0.11	0.14 ± 0.05
pCel-Tfu & pBHR68	Glucose	1.16 ± 0.09	5.91 ± 0.21	0.07 ± 0.01
	CMC	1.99 ± 0.13	2.57 ± 0.16	0.05 ± 0.01

When CMC was used as sole carbon source, the cellulolytic *E. coli* accumulated 1.99 g/L cell mass with a PHB yield of 0.05 g/L. The PHB yield in the control group was 0.07 g/L with a cell mass of 1.16 g/L (Table 1). Although the cell biomass was high in the CMC medium, the PHB content and yield was significantly lower than that using glucose as the carbon source. This indicated that the PHB accumulation needed a metabolic overflow caused by glucose excess while cell biomass accumulation depended on the total substrate amount.

### Investigating the secretion possibility of other hydrolytic enzymes

We further investigated whether Cel-CD and N20 could be used as the fusion partner for the extracellular production of other hydrolytic enzymes. Four hydrolytic enzymes from different sources were investigated, including pectate lyase C (PelC) from *Bacillus subtilis*, xylobiose (Xsa) from *Bacteroides ovatus* and two endoxylanases, XynC-A from *Fibrobacter succinogenes* and Xyn10B from *Clostridium stercorarium*, respectively. The secretion of these recombinant proteins was analyzed as shown in Table 2. All recombinant proteins were detected in the LB medium after 24 h of cultivation. However, the secretion levels varied.

**Table 2:** Secretion of other hydrolytic enzymes from different sources

Recombinant enzymes	Origin	Function	*Mw* (kDa)	Accession number	Secretion amount (mg/L)
N20-XynC-A	*F. succinogenes*	Endoxylanase	28.2	U01037	52.6 ± 7.2
N20-Xyn10B	*C. stercorarium*	Endoxylanase	44.3	AJ508407	53.9 ± 14.1
N20-PelC	*B. subtilis*	Pectate lyase	24.3	KIU05784	106.7 ± 9.4
Cel-PelC					264.6 ± 11.4
Cel-Xsa	*B. ovatus*	Xylobioase	38	AAB08024	104.2 ± 8.7

Fusion of Cel-CD with Xsa led to a relative high secretion as the extracellular accumulation of Cel-Xsa reached 104.20 mg/L. However the recombinant N20-xsa was not detected in the medium. Two endoxylanases XynC-A and Xyn10B were secreted in the medium when fused with N20. The extracellular proteins were 52.64 and 53.98 mg/L, respectively. Cel-XynC-A and Cel-Xyn10B were not secreted. Among these four hydrolytic enzymes, only PelC was detected in the medium regardless of fusion with Cel-CD or N20. The extracellular recombinant Cel-PelC reached 264.63 mg/L and the recombinant N20-pelC reached 106.72 mg/L. These results indicated that the Cel-CD and N20 can be used as a general carrier protein for the extracellular production of various hydrolytic enzymes.

## Discussion

Various strategies in engineering microorganisms for conversion plant biomass to biofuels and biorefinery products are being explored because of its great potential in reducing the production cost [23]. To engineer a cellulolytic *E. coli* strain, the common method is extracellular expression of cellulases by fusion with carrier proteins [24]. However, commonly used carrier proteins are not involved in cellulose hydrolysis [13]. In our previous study, we found the Cel-CD progressively accumulated in the medium and the final extracellular protein concentration reached 514 mg/L after 24 h of cultivation [22]. The high accumulation of cellulase amount suggested that this system could be used for the production of biochemicals and biofuels directly from cellulose. As the cellulase can be secreted in *E. coli* itself, cellulolytic *E. coli* can be constructed easily with minimum protein components.

In this study, the recombinant expression of only Cel-CD and β-glucosidase was enough to construct a cellulolytic *E. coli* successfully. The properties of Tfu0937 are well matched with *E. coli* culture conditions (pH and temperature), therefore it displayed good activity [25]. As the accumulation of cellobiose inhibits the action of endo- and exo-glucanases, the addition of β-glucosidase may alleviate the inhibition and improve their activity [26]. In our study, the fusion protein of Cel-Tfu may have a synergistic function that improves both endo-glucanase and β-glucosidase activities. The Cel-CD degradation product, oligosaccharides, may provide more substrates and accessibility for BGL, while the BGL generated product, glucose, can be consumed by the host and therefore may release the feedback inhibition on BGL and endo-glucanase.

Polyhydroxybutyrate (PHB) is a kind of biodegradable polyester that can be applied in many fields [27]. A major problem facing the commercialization of PHB is their much higher production cost compared with petrochemical based synthetic plastic materials [28, 29]. Using cellulose as carbon source, the production cost can be reduced. However, the overall PHB production yield was still low in this cellulolytic *E. coli*. This may because of two reasons: first, the *E. coli* strain BL21 (DE3) is not a suitable strain for PHB production. When the PHB accumulation in *E. coli* BL21 (DE3) was compared with that of DH5α under the same conditions, the PHB accumulation in *E. coli* BL21 (DE3) was two times lower than that of DH5α (data not shown). The reason of this result is not clear, but may be for the metabolic nature of BL21. Second, the only difference between the glucose control group of Cel-Tfu and N20-Tfu was the molecular weight of the expressed recombinant proteins. But the PHB yield of N20-Tfu was four times that of Cel-Tfu using glucose as a carbon source under the same conditions. We inferred that the overexpression of the recombinant protein in *E. coli* BL21 (DE3) may have a negative effect on PHB accumulation, because both the PHB granules aggregate and protein expression are intracellular. Nevertheless, this is the first report that PHB was produced in a constructed cellulolytic *E. coli*. This process can be adapted to produce other biochemicals and biofuels directly from plant biomass.

We also investigated whether Cel-CD and N20 could be used as the fusion partners for the extracellular production of other hydrolytic enzymes. Xylan is a representative hemicellulose, characterized by a β-1,4-linked xylose backbone [30]. The secretion of xylanase and xylobiose fusion with Cel-CD offered a platform for the conversion of xylan, which is an abundant renewable biomass, by *E. coli*. Meanwhile, pectate lyase can be secreted by fusion with both Cel-CD and N20, indicating that pectin hydrolytic recombinant *E. coli* can be constructed. However, some investigated enzymes still cannot be secreted by fusion with Cel-CD or its N-terminal sequence (data not shown). The intrinsic mechanism underlying this will be investigated in a subsequent study.

## Conclusions

The *E. coli* strains are commonly used as host for the industrial production of various chemical products. However, it is unable to degrade cellulose polymers because lack of endogenous cellulases. In this study, a catalytic domain of a cellulase (Cel-CD) and its N-terminus were both employed as carriers for the extracellular production of a β-glucosidase (Tfu0937) in *E. coli* BL21 (DE3). The fusion proteins digested the cellulose outside the host and the resulted glucose can be imported into the cells. Based on this Cel-secretion system, we set up a PHB production bioprocessing in *E. coli* strain using cellulose as the sole carbon source.

## Methods

### Bacterial strains and plasmids construction

The strains and plasmids used in this study are shown in Table 3. *E. coli* strain DH5α (Invitrogen, USA) was used for recombinant DNA manipulation. *E. coli* BL21 (DE3) (Novagen, Germany) was used as the host for the expression of Cel-CD and other recombinant proteins. Antibiotics were added as follows: chloromycetin (Cm) at 34 μg/mL.

**Table 3:** The strains, plasmids and primers used in this study

Description	Description	Source
Strains		
*E. coli* DH5α	F^−^ *endA1 hsdR17(r*_*K*_− *m*_*K*_+*) supE44 thi*-*l λ* ^−^ *recA1 gyrA96 ΔlacU169 (Φ80dlacZM15*)	Invitrogen
*E. coli* BL21(DE3)	*hsdS, gal(ΔcIts857 ind1, Sam7, nin5, lacUV5*-*T7, gene1*	Novagen
Plasmids		
pACYCDuet	4.0 kb, p15A ori, T7 promoter, Cm^R^	Novagen
pACYCDuet/cel	pACYCDuet, *cel*-*cd* gene under T7-1 promoter	This study
pACYCDuet/n20	pACYCDuet, *n20* gene under T7-1 promoter	This study
pCel-Tfu	*cel*-*cd* gene fusion with *tfu* from *Thermobifida fusc*a	This study
pN20-Tfu	*n20* gene fusion with *tfu* from *Thermobifida fusc*a	This study
pBHR68	pSK-derivative, *phbA*, *phbB* and *phbC* from *Ralstroniaeutropha*	[31]
pN20XynC-A	*n20* gene fusion with *xynC* from *F. succinogenes*	This study
pN20Xyn10B	*n20* gene fusion with *xyn10b* from *C. stercorarium*	This study
pN20PelC	*n20* gene fusion with *pelC* from *B. subtilis*	This study
pCelPelC	*cel*-*cd* gene fusion with *pelC* from *B. subtilis*	This study
pCelXsa	*cel*-*cd* gene fusion with *xsa* from *B. ovatus*	This study

Primers	Sequences
Cel-*Nco*I-F	5′- TTTTCCATGGAAGGAAACACTCGTGAAGA-3′
Cel-*Bam*HI-R	5′- TTTTGGATCCAAGTACTTTCGTGTATTTTG-3′
Cel-20-*Bam*HI-R	5′- TTTTGGATCCGCGTTTAACATTGTCATTAC-3′
Tfu-*Bam*HI-F	5′-TTTTGGATCCACCTCGCAATCAACGACGCC-3′
Tfu-*Xho*I-R	5′-TTTTCTCGAGTTATTCTTGACCGAAAATACCAC-3′
XynC-A-*Bam*HI-F	5′- TTTTGGATCC CAAGACTTCTGCAGCAACGC-3′
XynC-A-*Xho*I-R	5′-TTTTCTCGAGGTTTTTAACATAAACTTTCG-3′
Xyn10B-*Bam*HI-F	5′- TTTTGGATCCAACAAGTTCCTGAACAAGAA-3′
Xyn10B-*Xho*I-R	5′-TTTTCTCGAGTTCGCGCAGACGAGACGGAT-3′
PelC-*Bam*HI-F	5′- TTTTGGATCCGCAGATAAGGTTGTCCACGA-3′
PelC-*Xho*I-R	5′- TTTTCTCGAGTTAGAACTGGGTGTTATTAT-3′
Xsa-*Bam*HI-F	5′- TTTTGGATCCAAGACCGAAAAGCGTTACCT-3′
Xsa-*Xho*I-R	5′-TTTTCTCGAGTTCGTCTTTGCCTTCAATGG-3′

The Cel-CD expression plasmid was created based on pACYCDuet (Novagen). The DNA sequence encoding the Cel-CD (Accession: M84963, 1494 to 2618) used in this study was amplified from the genome of *Bacillus* sp. (lab collection) by polymerase chain reaction using PrimeSTAR (TaKaRa) with the Cel-*Nco*I-F and Cel-*Bam*HI-R as primers. After digestion with *Nco*I and *Bam*HI (Fermentas), the amplified DNA fragment was inserted into pACYCDuet using T4 DNA ligase (NEB), generating plasmid pACYCDuet/cel. The DNA sequence encoding N20 amino acid residues of Cel-CD used in this study was amplified from the plasmids pACYCDuet/cel by polymerase chain reaction using PrimeSTAR (TaKaRa) with the Cel-*Nco*I-F and Cel-20-*Bam*HI-R as primers to construct pACYCDuet/n20.

To fuse the *tfu0937* gene (Accession: AAF37730) to the 3′ end of *cel*-*cd*, the DNA fragments were amplified by PCR, and digested with restriction enzymes *Bam*HI and *Xho*I on both ends. The resulting fragments were then ligated to the vector pACYCDuet/cel by T4 DNA ligase to obtain the objective plasmids. The same method was used to construct other N20 and Cel-CD fusion proteins.

### Culture conditions

During DNA manipulations and recombinant protein secretion tests, cultures were grown at 37 °C in Luria Bertini (LB) medium (10 g/l tryptone, 5 g/l yeast extract, and 10 g/l NaCl, pH 7.2). For PHB accumulation, cells were cultured in 5 mL LB medium for 12 h at 37 °C. The cells were collected, washed three times and inoculated in modified M9 medium (Na_2_HPO_4_, 15.138 g/L; KH_2_PO_4_, 3 g/L; NaCl, 0.5 g/L; NH_4_Cl, 1 g/L; yeast, 2 g/L) supplemented with a different carbon source. To induce the expression of the desired enzymes, isopropyl-β-D-thiogalactopyranoside (IPTG) was added to a final concentration of 0.5 mM when OD_600_ reached 0.8. After induction, cells were cultured until the indicated time.

### Cel-CD activity assay

The hydrolytic activity of the secreted Cel-CD was determined by the Dinitrosalicylic acid (DNS) method [32]. Culture medium (0.5 mL) was added to 2 mL of Britton-Robinson buffer containing 1 % CMC-Na in a test tube. The reaction mixture was incubated at 37 °C for 15 min, then 2 mL DNS reagent was added and the mixture was boiled for 7 min before it was diluted to a final volume of 12.5 mL. The enzyme activity was measured with a spectrophotometer at 540 nm.

### Tfu0937 activity assay

The hydrolytic activity of the secreted Tfu0937 was determined by the p-nitrophenol-β-d-glucopyranoside (pNPG) method [33]. A reaction mixture (1 mL) containing 750 μL of culture medium and 250 μL of pNPG (2 mM) as the substrate was incubated at 37 °C for 15 min, and the reaction was terminated by adding 200 μL of 2 mM NaOH. The developed yellow color was read at 410 nm, and the enzyme activity was quantified using the pNPG standard. One unit of β-glucosidase activity was expressed as the amount of enzyme required to release 1 μmoL of pNPG per minute under the assay conditions.

### Congo red staining

To determine the hydrolytic activity of Cel-CD, an agarose plate based assay was used [34]. 1 % (wt/vol) carboxymethylcellulose (CMC-Na) was added in the agarose plate as a substrate (0.8 %, wt/vol). Samples containing the Cel-CD were loaded into the plate hole. The plate was incubated at 30 °C for 3 h, then flooded with 0.1 % (wt/vol) Congo red solution and stained for 15 min at room temperature. The dye was removed, and 5 mL of water was used to wash the plate. Finally, 5 mL 0.9 % NaCl solution was applied for 15 min, and the plates were then dried and photographed.

### SDS-PAGE

Cells were harvested at the indicated time by centrifugation after induction and washed once with 1 mL of 100 mM 3-(*N*-morpholino)propanesulfonic acid (MOPS) (pH 7.0). The washed cell pellets were then disrupted by ultrasonication and centrifuged at 10,000*g* for 15 min to obtain the total soluble protein samples. The secreted protein samples were obtained directly by centrifugation. The samples were then prepared by adding an equal volume of electrophoresis loading buffer. Protein samples were separated on 12 % SDS-PAGE.

### Gas chromatography analysis

PHB production yields were determined by gas chromatography (GC). Cells were harvested by centrifugation at 10,000*g* for 3 min. The cell pellets were washed with distilled water twice and then lyophilized for 6 h. Before GC analysis, a 15–20 mg sample of dried cells was treated with 1 mL chloroform, 850 μL methanol, and 150 μL sulfuric acid (98 %, w/w), which were added to the weighed cells. The vials were incubated at 100 °C for 1 h. Then, 1 mL water was added to the cooled vials. After phage separation, the heavier chloroform phase was transferred to another new vial for GC analysis. The GC detection process was performed according to the method of [35] using a Shimadzu GC2010 gas chromatograph (Kyoto, Japan) equipped with an AOC-20i auto injector and a Restek Rtx-5 column.
